# XBB.1.5 spike protein COVID-19 vaccine induces broadly neutralizing and cellular immune responses against EG.5.1 and emerging XBB variants

**DOI:** 10.1038/s41598-023-46025-y

**Published:** 2023-11-06

**Authors:** Nita Patel, Jessica F. Trost, Mimi Guebre-Xabier, Haixia Zhou, Jim Norton, Desheng Jiang, Zhaohui Cai, Mingzhu Zhu, Anthony M. Marchese, Ann M. Greene, Raburn M. Mallory, Raj Kalkeri, Filip Dubovsky, Gale Smith

**Affiliations:** Novavax, Inc., Gaithersburg, MD USA

**Keywords:** Immunology, Microbiology

## Abstract

Monovalent SARS-CoV-2 Prototype (Wuhan-Hu-1) and bivalent (Prototype + BA.4/5) COVID-19 vaccines have demonstrated a waning of vaccine-mediated immunity highlighted by lower neutralizing antibody responses against SARS-CoV-2 Omicron XBB sub-variants. The reduction of humoral immunity due to the rapid evolution of SARS-CoV-2 has signaled the need for an update to vaccine composition. A strain change for all authorized/approved vaccines to a monovalent composition with Omicron subvariant XBB.1.5 has been supported by the WHO, EMA, and FDA. Here, we demonstrate that immunization with a monovalent recombinant spike protein COVID-19 vaccine (Novavax, Inc.) based on the subvariant XBB.1.5 induces neutralizing antibodies against XBB.1.5, XBB.1.16, XBB.2.3, EG.5.1, and XBB.1.16.6 subvariants, promotes higher pseudovirus neutralizing antibody titers than bivalent (Prototype + XBB.1.5) vaccine, induces SARS-CoV-2 spike-specific Th1-biased CD4 + T-cell responses against XBB subvariants, and robustly boosts antibody responses in mice and nonhuman primates primed with a variety of monovalent and bivalent vaccines. Together, these data support updating the Novavax vaccine to a monovalent XBB.1.5 formulation for the 2023–2024 COVID-19 vaccination campaign.

## Introduction

Prior to recommendations for COVID-19 vaccine strain change and composition harmonization, COVID-19 vaccine options included monovalent SARS-CoV-2 Prototype (Wuhan-Hu-1) and bivalent (Prototype + Omicron BA.4/5). The need to update the vaccine strain has been signaled by a reduction of antibody-mediated immunity against Omicron XBB subvariants^1^. A vaccine strain and composition change to monovalent XBB.1.5-based vaccines have been supported by the World Health Organization (WHO), the European Medicines Agency (EMA), and the United States Food and Drug Administration (FDA) in anticipation of the Fall 2023–2024 booster campaigns^2–4^. This change in vaccine composition highlights the need for robust immunologic evaluation of emerging Omicron XBB sub-variants.

The Novavax COVID vaccine platform contains the full-length recombinant spike (rS) protein presented in its native trimeric and prefusion conformation^5,6^. Spike protein trimers form particles surrounding a polysorbate-80 core, which may improve antigen uptake and processing. Protein antigens are manufactured in a baculovirus expression system using *Spodoptera frugiperda* moth cells (Sf9) that express glycoproteins with truncated N-linked glycans with a potential for enhanced epitope exposure. Proteins expressed in Sf9 insect cells are post-translationally modified with smaller and less processed N-linked glycans, helping critical epitopes for antibody binding remain exposed compared to proteins produced in mammalian cells^7^. Antigen particles are formulated with a saponin-based Matrix-M™ adjuvant^8^, which has been shown to induce robust antibody responses that protect upper and lower airways in nonhuman primates^9^ and polyfunctional Th1-biased CD4 + T cell responses^10–12^. NVX-CoV2373, based on the Prototype (Wuhan-Hu-1) rS, was demonstrated to be well tolerated and immunogenic in humans^12,13^, with a vaccine efficacy of 90.4% (95% CI; 82.9 to 94.6) against mild-to-severe disease in a Phase 3 clinical trial^14^. NVX-CoV2373 has been authorized for primary series use and as a homologous or heterologous booster in many countries globally.

In 2022, the United States FDA and many global regulators authorized updated bivalent mRNA vaccine boosters containing sequences of both the ancestral and Omicron BA.1 or Omicron BA.4/BA.5 spike proteins. Since the addition of bivalent vaccines, their benefits, compared to monovalent options, have been debated. It may be that the presence of Prototype spike in the current bivalent vaccines leads to original antigenic sin, also known as immunological imprinting, that can bias immune responses to the development of lower immunity to the variant spike^15^.

Omicron XBB lineages likely emerged following a recombination of two co-circulating Omicron BA.2 lineages, BJ.1 and BM.1.1.1, during the summer of 2022^16^. At the time of vaccine strain recommendations by the WHO, EMA, and FDA, Omicron XBB.1.5 represented a well characterized strain among the currently prevalent and emergent strains. Sequence comparison supported XBB.1.5 as the preferred vaccine strain due to sequence similarity to other emerging XBB variants. XBB.1.16 spike has two amino acid differences (E180V and T478R) from XBB.1.5^17^. XBB.2.3 has notable amino acid differences (V252G, D253G, and P521S) compared to XBB.1.5, and from XBB.1.16 (G252V, D253G, T478K, P521S). A descendant lineage of XBB.1.9.2, EG.5, has an additional spike amino acid difference (F456L) compared to XBB.1.5. Subvariant EG.5.1, which has an additional spike amino acid difference (Q52H), has become prevalent in some regions of the world^18^. BA.2.86 has > 35 amino acid differences compared with XBB.1.5^19^. Due to the continued rapid evolution of the SARS-CoV-2 virus, the ability of the updated vaccines to generate cross-protective immunity to future viral variants will be critical as a periodic COVID-19 vaccine strain-change has been suggested. Preclinical data from animal models are needed to inform and support the update of the next-generation COVID-19 vaccine composition to a monovalent XBB.1.5 vaccine for the 2023–2024 vaccination season.

## Results

### Primary immunization with prototype, bivalent, XBB.1.5, or XBB.1.16 rS in mice

**Figure 1:**
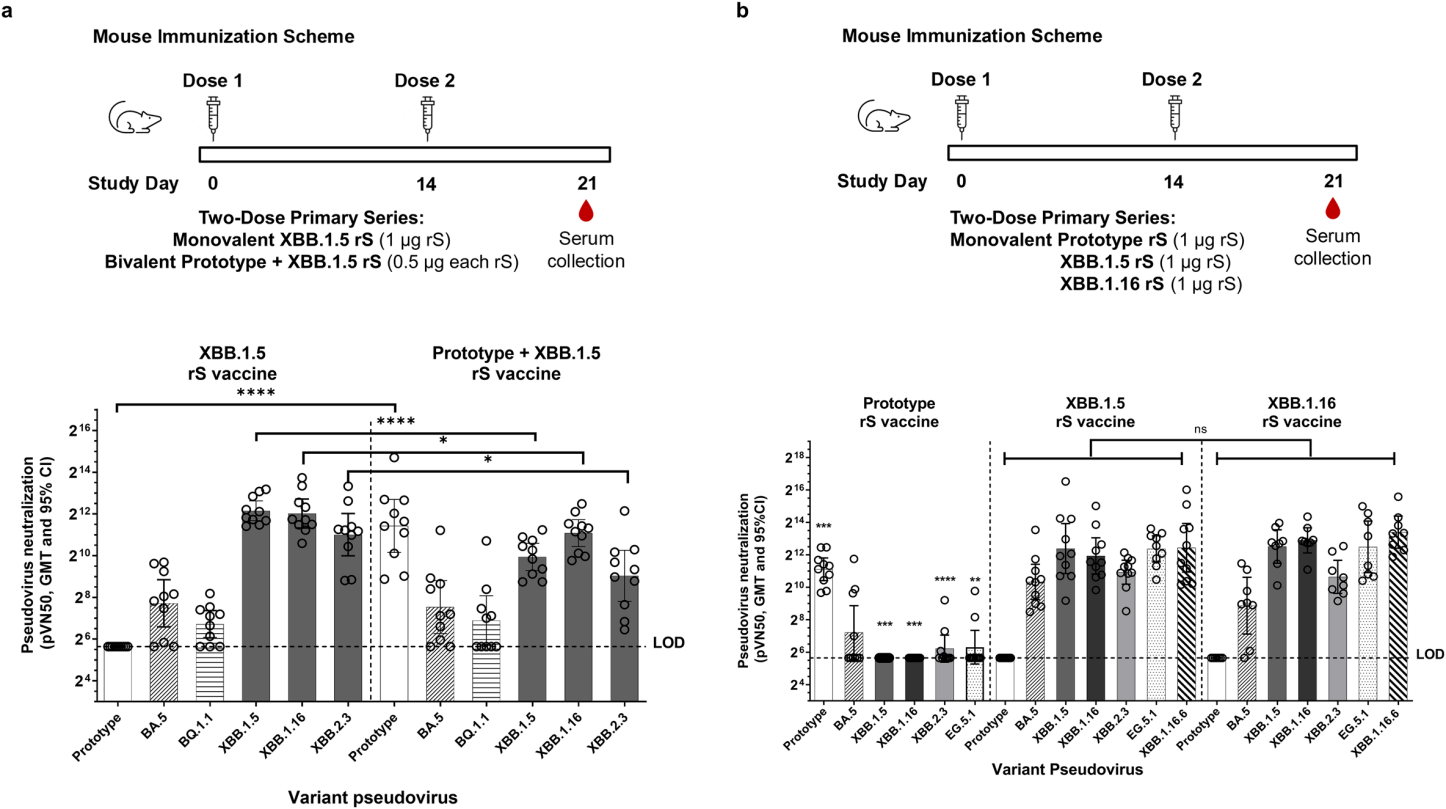
Humoral responses following variant-adapted two-dose primary series vaccination in mice. (**a**) Pseudovirus neutralization in mice sera collected one week following primary vaccination with two doses of monovalent XBB.1.5 or bivalent (Prototype + XBB.1.5). Sera were collected on Day 21 as indicated in the study design diagram. (**b**) Pseudovirus neutralization in mice sera collected one week following primary series vaccination with two doses of monovalent Prototype, XBB.1.5, or XBB.1.16. Sera were collected on Day 21 as indicated in the study design diagram. Note that all immunizations were administered with 5 µg Matrix-M adjuvant. Open circles represent individual data points, solid bars represent group geometric mean titers, error bars represent 95% confidence intervals, and the horizontal dashed line represents the assay limit of detection (LOD). Statistically significant differences are marked with asterisks: *P < 0.05; **P < 0.01; ***P < 0.001; ****P < 0.0001. In Panel (**b**), asterisks represent differences that were significant between the Prototype rS group and the other two immunization groups.

We assessed humoral immune responses in female BALB/c mice following two-dose primary series immunization with monovalent and bivalent vaccines. Mice (n = 10 per group) were inoculated intramuscularly with XBB.1.5 (1 µg rS) or bivalent rS (0.5 µg Prototype rS + 0.5 µg XBB.1.5 rS) on days 0 and 14, and sera were collected at day 21 (1 week after the second dose). The monovalent XBB.1.5 rS vaccine resulted in higher neutralizing titers against more recently emerged variants of concern XBB.1.5 (*P* < 0.0001), XBB.1.16 (*P* = 0.043), and XBB.2.3 (*P* = 0.015) pseudoviruses, compared to a bivalent approach (Fig. 1a). Immunization with the bivalent Prototype rS + BA.5 rS resulted in statistically significantly higher neutralizing titers against Prototype pseudovirus (*P* < 0.0001), but since this strain is no longer circulating, this would not confer any practical protection.

We next compared the induction of pseudovirus neutralizing antibodies after immunization with a primary series of various monovalent rS constructs. Groups of mice (n = 10) were inoculated intramuscularly with Prototype, XBB.1.5, or XBB.1.16 rS on days 0 and 14, and sera were collected at day 21 (1 week after the second dose). Humoral responses following XBB.1.5 or XBB.1.16 primary series were highly immunogenic against homologous strains and other contemporary variants, as they induced pseudovirus neutralizing antibodies against SARS-CoV-2 Omicron BA.5, XBB.1.5, XBB.1.16, XBB.2.3, EG.5.1, and XBB.1.16.6, with no significant differences between these two groups (Fig. 1b). Immunization with the two-dose primary series of Prototype rS resulted in statistically significantly lower antibody titers against XBB.1.5, XBB.1.16, XBB.2.3, and EG.5.1 than those observed after a primary series with either XBB.1.5 rS or XBB.1.16 rS, emphasizing the crucial need for updated vaccines to provide antibody coverage against contemporary SARS-CoV-2 variants of concern (Fig. 1b).

### XBB lineage booster immunization in mice and nonhuman primates

**Figure 2:**
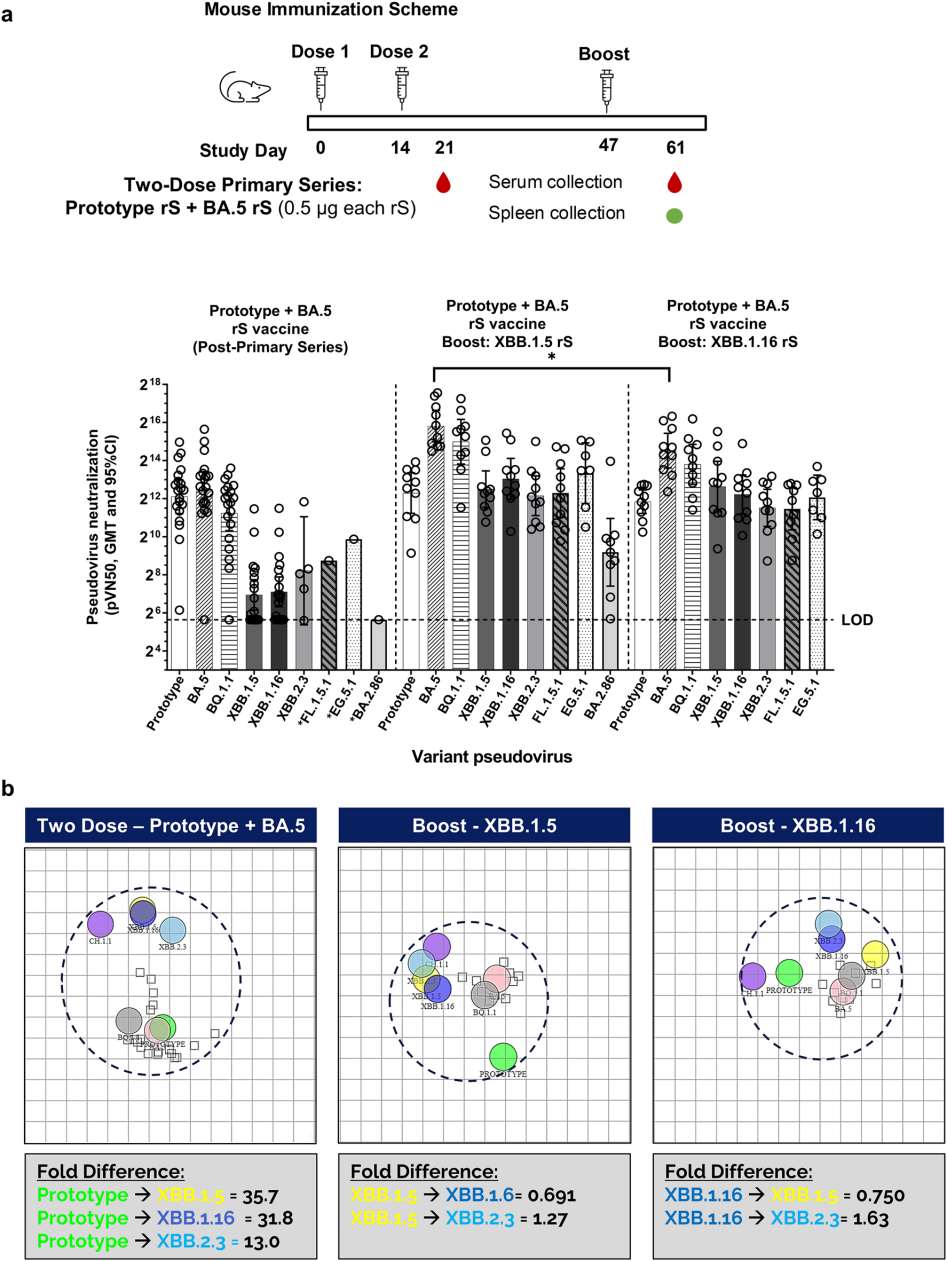
Humoral responses following XBB.1.5 booster in mice. (**a**) Pseudovirus neutralization titers were determined in mouse sera collected one week after a primary series with bivalent Prototype rS + BA.5 rS (left graph series), and following a boost with monovalent XBB.1.5 (middle graph series) or XBB.1.16 (right graph series) vaccines (sera collected two weeks after booster dose). Note that all immunizations were administered with 5 µg Matrix-M adjuvant. Open circles represent individual data points, solid bars represent group geometric mean titers, error bars represent 95% confidence intervals, and the horizontal dashed line represents the assay limit of detection (LOD). Statistically significant differences between the two booster groups are marked with asterisks: *P < 0.05. Pooled sera were analyzed for Day 21 data where indicated with an asterisk on the x-axis label. (**b**) Pseudovirus neutralizing titers presented in (**a**) were subjected to antigenic cartography analysis. Each small square corresponds to one animal and each grid square corresponds to one antigenic distance of twofold change in neutralization titer with fold differences given below each map. Smaller antigenic distance fold-change between two variants indicates higher cross-neutralizing antibody titers.

We also investigated immune responses in mice primed with bivalent rS (Prototype rS + BA.5 rS) followed by a booster dose of monovalent XBB.1.5 or XBB.1.16. Groups of mice (n = 10 per group) were inoculated intramuscularly with a primary immunization series of a bivalent (Prototype rS + BA.5 rS) vaccine on days 0 and 14, followed by a single booster dose with XBB.1.5 rS or XBB.1.16 rS on day 47. Sera were collected at day 21 (1 week after the second dose) and day 61 (2 weeks after booster dose). XBB.1.5 and XBB.1.16 induced a > 35-fold increase in pseudovirus neutralizing antibodies against XBB.1.5 and XBB.1.16 compared to the titers after the primary series (Fig. 2a). For all variant pseudoviruses examined, including forward drifted variants FL.1.5.1 and EG.5.1, neutralizing titers were not statistically significantly different after a booster with either XBB.1.5 rS or XBB.1.16 rS, with the exception of Omicron BA.5, for which the booster with XBB.1.5 rS resulted in higher antibody titers (Fig. 2a). A booster dose with XBB.1.5 rS resulted in detectable antibody titers against BA.2.86, which were undetectable after the primary series with bivalent Prototype rS + BA.5 rS. Pseudovirus neutralizing antibody titers in mice were further analyzed by antigenic cartography, a method for visualizing antigenic diversity. Priming with two doses of bivalent vaccine (Prototype + BA.5) resulted in greater than 30-fold differences in neutralizing responses between Prototype to both XBB.1.5 and XBB.1.16 shown by antigenic cartography (Fig. 2b). This large antigenic distance was expected as the neutralizing epitopes of Prototype and BA.5 rS are mainly absent on XBB sub-variants. Antigenic distances with a fold-difference less than twofold are considered to be matched responses. Boosting primed mice with XBB.1.5 vaccine induced a matched response to XBB.1.16, with an antigenic distance of 0.691 (Fig. 2b). Similarly, an XBB.1.16 boost induced a matched response to XBB.1.5 with a fold-difference of 0.750 (Fig. 2b).

**Figure 3:**
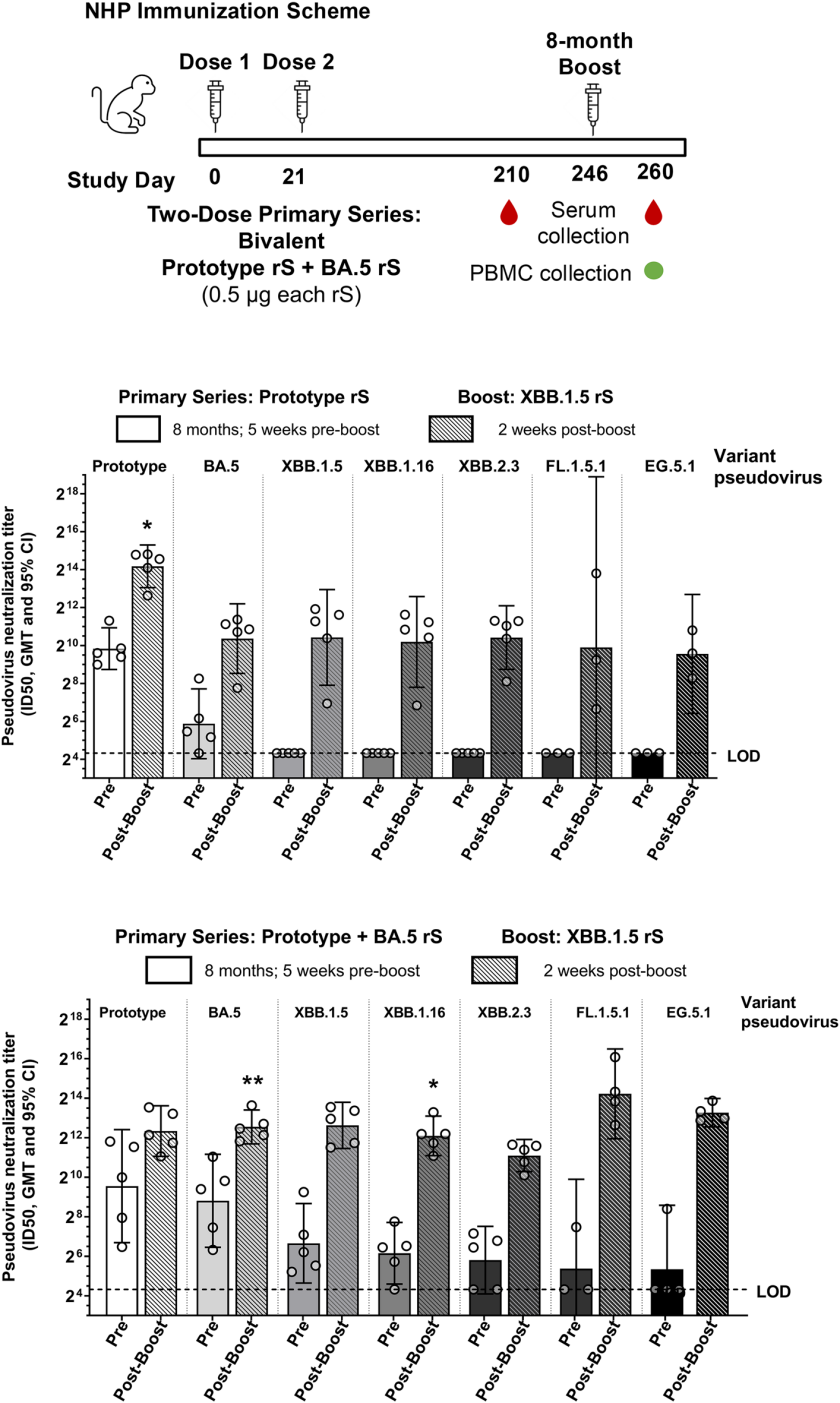
Humoral Responses Following XBB.1.5 Booster in Rhesus Macaques. Pseudovirus neutralization titers in rhesus macaques boosted with XBB.1.5 approximately 8 months after Prototype rS (top graph) or bivalent (Prototype + BA.5; bottom graph) priming regimens as shown in the study design diagram. Note that all immunizations were administered with 50 µg Matrix-M adjuvant. Sera were collected five weeks before the boost (Day 210) and two weeks after the booster dose (Day 260). Statistically significant differences in post-boost titers between the two groups after are marked with asterisks above the group with the higher titers: *P < 0.05; **P < 0.01. Open circles represent individual data points, solid bars represent group geometric mean titers, error bars represent 95% confidence intervals, and the horizontal dashed line represents the assay limit of detection.

The impact of priming series immunization strain and composition on XBB.1.5 pseudovirus neutralization was also tested in nonhuman primates (NHPs). Rhesus macaques (*Macaca mulatta*; n = 5 per group) received a two-dose primary series of either Prototype or bivalent (Prototype + BA.5) vaccines and were boosted with XBB.1.5. All NHPs were monitored twice daily to determine the safety of the vaccines, and no local or systemic side effects were reported after the primary series or booster vaccinations. For both priming regimens, boosting with XBB.1.5 increased neutralizing antibody titers against XBB.1.5, XBB.1.16, XBB.2.3, FL.1.5.1, and EG.5.1 pseudoviruses by 39.2- to 243.5-fold compared to pre-boost titers (Fig. 3). The bivalent primary series regimen containing BA.5 resulted in statistically significantly higher post-boost titers against BA.5 and XBB.1.16 compared to post-boost titers in NHPs that received a primary series of monovalent Prototype rS, though post-boost titers against XBB.1.5, XBB.2.3, FL.1.5.1, and EG.5.1 were not statistically significantly different, regardless of which primary series was administered (Fig. 3).

### CD4 + T cell responses in mice and nonhuman primates

**Figure 4:**
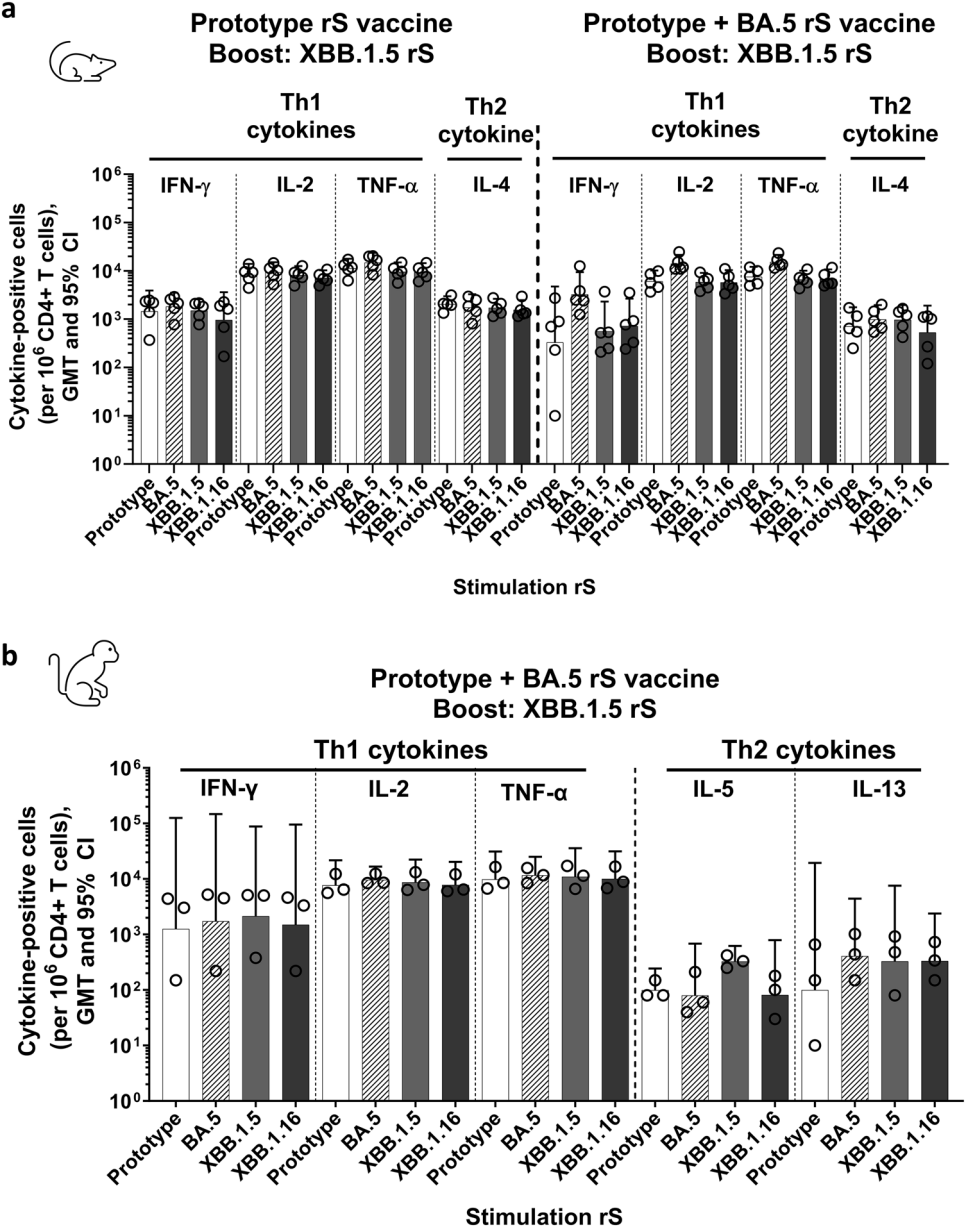
CD4 + T Cell Responses to an XBB.1.5 Booster. (**a**) CD4 + T cell responses in mice primed with two doses of Prototype or bivalent (Prototype + BA.5) rS and boosted with XBB.1.5 rS as outlined in Fig. 2. Splenocytes were collected two weeks after the booster dose. (**b**) CD4 + T cell responses in rhesus macaques primed with bivalent (Prototype + BA.5) rS and boosted with XBB.1.5 rS. Peripheral blood mononuclear cells (PBMCs) were collected 2 weeks after the booster dose. Open circles represent individual animal data points, solid bars represent group geometric mean values with 95% CI error bars.

To investigate the cellular responses, we measured CD4 + T cell responses in mice (n = 5 per group) immunized with Prototype or bivalent (Prototype + BA.5) priming series vaccine and boosted with XBB.1.5. Th1 (IFN-γ, IL-2, and TNF-α) and Th2 (IL-4) cytokine-producing CD4 + T cells were measured in splenocytes isolated two weeks post booster dose. A robust CD4 + T cell response was recalled at comparable levels post-boost upon stimulation with rS of XBB.1.5, XBB.1.16, or other variants, irrespective of priming vaccine (Fig. 4a). Similarly, NHPs primed with bivalent (Prototype + BA.5) vaccine and boosted with XBB.1.5 elicited a Th1-biased cellular response with comparable magnitudes of cytokine-positive cells for all variants tested (Fig. 4b).

## Discussion

Neutralizing antibodies inhibit viral infections by binding viral surface components that participate in host-cell fusion and entry. The generation of neutralizing antibodies specific for the envelope embedded SARS-CoV-2 spike glycoprotein following infection or vaccination is a crucial part of a functional SARS-CoV-2 immune response. Neutralizing antibodies can also be effective when used as prophylactic or therapeutic treatments against COVID-19^20^. The Novavax monovalent XBB.1.5 vaccine induced neutralizing responses against SARS-CoV-2 Omicron XBB lineage sub-variants including XBB.1.5, XBB.1.16, XBB.2.3, EG.5.1, XBB.1.16.6, and BA.2.86. In mice, a primary series of monovalent 1 µg XBB.1.5 rS generated higher-titer neutralizing responses than the bivalent vaccine composed of 0.5 µg each of Prototype and BA.5, suggesting an advantage to a monovalent vaccine formulation. This was likely due to the half a dose of the antigen (0.5 µg of each antigen in the bivalent vaccine compared to 1 µg of antigen in the monovalent vaccine) as there was no indication that original immune imprinting was associated with boosting. When administered as a booster dose in mice, monovalent XBB.1.5 and XBB.1.16 vaccines exhibited comparable pseudovirus neutralizing antibody responses against contemporary variants including XBB.1.5, XBB.1.16, XBB.2.3, FL.1.5.1, and EG.5.1. In nonhuman primates, the XBB.1.5 booster was shown to induce similar neutralizing responses against XBB.1.5, XBB.2.3, and EG.5.1 in animals primed with monovalent Prototype, or bivalent (Prototype + BA.5) vaccine. Considering that higher levels of neutralizing antibodies generally correlate with enhanced protection from disease and durability of immune responses^21–27^, the robust pseudovirus neutralizing antibody titers generated after immunization with XBB.1.5 rS vaccine observed in these preclinical studies will likely translate to favorable responses in the clinic against contemporary SARS-CoV-2 variants of concern.

The cell-mediated immunogenicity of the monovalent XBB.1.5 vaccine was further evaluated by measuring cell-mediated immune responses, which showed the presence of a polyfunctional Th1-biased CD4+ response against XBB sub-variants in mice and NHPs.

The contribution and relative importance of other mechanisms of immunity, such as non-neutralizing antibodies, opsonization, antibody-dependent cellular cytotoxicity, antibody-dependent cellular phagocytosis, and antibody-dependent complement deposition must be further explored (reviewed by Goldblatt et al.^28^). Population-level immunity against severe disease and hospitalization has persisted against emerging SARS-CoV-2 variants, despite evidence of reduced levels of vaccine-mediated variant neutralization^29^. Notably, Th1 cytokine signaling can promote the cytotoxic activities of CD8 + T cells and macrophages to destroy infected cells and limit the severity of disease. In addition, Fc-effector functional antibodies induced by NVX-CoV2373 were identified as key determinants of protection against infection in rhesus macaques and humans^30^. Though little focus has been placed on data describing cellular immunity or Fc-effector profiles, their role in a productive adaptive immune response should not be ignored. To our knowledge, this is the first booster study in a non-human primate (NHP) model for evaluating XBB.1.5 booster immunogenicity. This NHP booster immunogenicity study recapitulated the results from the mouse model studies that an XBB.1.5 booster produces strong neutralizing responses against the homologous antigen XBB.1.5, plus cross-neutralizing antibodies against XBB.1.16, XBB.2.3, FL.1.5.1, and EG.5.1 variants. Importantly, the XBB.1.5 booster was immunogenic irrespective of priming regimen, as the general population includes individuals primed with diverse vaccination and infection backgrounds. Together, these data demonstrate that a next-generation Novavax COVID-19 vaccine based on monovalent XBB.1.5 rS can induce robust humoral and cellular immunity to EG.5.1, FL.1.5.1, BA.2.86, and XBB sub-variants (XBB.1.5, XBB.2.3, and XBB.1.16.6) in mice and NHPs primed with Prototype and bivalent vaccines. Cross-neutralization of the immune response generated by XBB.1.5 across EG.5.1, BA.2.86 and other XBB variants (XBB.1.16, XBB.2.3, and XBB.1.16.6) is also encouraging, in addressing emergence of either forward drifted strains. Consistent with recommendations by the WHO, EMA, and FDA, our preclinical data in mice and non-human primates support updating the Novavax vaccine to a monovalent XBB.1.5 formulation for the 2023–2024 COVID-19 season.

## Methods

### Vaccine constructs

SARS-CoV-2 Prototype rS (construct BV2373) was manufactured by the Novavax Discovery Group (Gaithersburg, MD). The SARS-CoV-2 rS vaccine is constructed from the full-length, wild-type SARS-CoV-2 S glycoprotein, based upon the GenBank gene sequence MN90894, nucleotides 21,563–25,384 (SARS-CoV-2 Wuhan-Hu-1 variant). The native full-length S protein was modified by mutation of the putative furin cleavage site RRAR to QQAQ (3Q) located within the S1/S2 cleavage domain to be protease resistant. Two additional proline amino acid substitutions were inserted at positions K986P and V987P (2P) within the heptad repeat 1 (HR1) domain to stabilize SARS-CoV-2 S in a prefusion conformation, which is believed to optimize presentation of neutralizing epitopes^31^.

SARS-CoV-2 Omicron BA.5. XBB.1.5 rS, and XBB.1.16 (constructs BV2540, BV2601, and BV2633), based on the Omicron BA.5, XBB.1.5, and XBB.1.16 variants of SARS-CoV-2, were manufactured by the Novavax Discovery Group (Gaithersburg, MD). Omicron BA.5, XBB.1.5, and XBB.1.16 variant sequences were obtained from the GISAID database references EPI-ISL 12,097,410.1, 16,343,574, and 17,351,426. To produce construct BV2540, the native full-length S protein was subjected to mutations applied to the Prototype Wuhan-Hu-1 rS plus additional mutations: V3G, T19I, A27S, G142D, V213G, G339D, S371F, S373P, S375F, T376A, D405N, R408S, K417N, N440K, L452R, S477N, T478K, E484A, F486V, Q498R, N501Y, Y505H, D614G, H655Y, N679K, P681H, N764K, D796Y, Q954H, and N969K, as well as ∆L24, ∆P25, ∆P26, ∆H69, and ∆V70. To produce construct BV2601, in addition to the mutations applied to the Prototype Wuhan-Hu-1 rS, the following mutations were introduced to the native full-length S protein: T19I, A27S, V83A, G142D, H146Q, Q183E, V213E, G252V, G339H, R346T, L368I, S371F, S373P, S375F, T376A, D405N, R408S, K417N, N440K, V445P, G446S, N460K, S477N, T478K, E484A, F486P, F490S, Q498R, N501Y, Y505H, D614G, H655Y, N679K, P681H, N764K, D796Y, Q954H, and N969K, as well as ∆24–26 and ∆Y144. To produce construct BV2633, in addition to the mutations applied to the Prototype Wuhan-Hu-1 rS the following mutations were introduced to the native full-length S protein: K986P, V987P, E180V, K478R from the BV2601 construct. Altogether the matured form of XBB.1.5, XBB.1.16, variant rS are 1255 amino acids due to the deletions at amino acids 24–27 and 144; BA.5 rS is 1256 amino acids due to deletions in the N-terminal domain at position 24–27 and 69–70 relative to the Prototype BV2373. The expected molecular mass of the glycosylated spikes variant proteins is ~ 161,707 Daltons. Recombinant baculoviruses were cloned and rS expressed in Sf9 insect cells and purified as described previously^10^.

### Animal ethics statement

The reporting in this manuscript follows the recommendations in the ARRIVE guidelines.

The mouse studies were conducted at Noble Life Sciences (Sykesville, MD). Animals were maintained and treated according to Animal Welfare Act Regulations, the US Public Health Service Office of Laboratory Animal Welfare Policy on Humane Care and Use of Laboratory Animals, Guide for Care and Use of Laboratory Animals (Institute of Laboratory Animal Resources, Commission on Life Sciences, National Research Council, 1996), and AAALACi accreditation. Mouse studies were approved by Noble Life Sciences IACUC.

The study in rhesus macaques was conducted at Texas Biomedical Research Institute (San Antonio, TX). Animals were maintained at Texas Biomedical Research Institute for the entire in-life portion of the study and were treated according to Animal Welfare Act regulations and the Guide for the Care and Use of Laboratory Animals (2011). Rhesus macaque studies were approved by Texas Biomedical Research Institute IACUC.

### Mouse study designs

For the primary series studies, female BALB/c mice (10–12 weeks old, weight range 17–22 g, N = 10–20 per group, total 80–100 per study) were immunized by intramuscular (IM) injection with two 1 µg doses spaced 14 days apart (study day 0 and 14) of monovalent Prototype (control), XBB.1.5, XBB.1.16, or bivalent Prototype + XBB.1.5 (0.5 µg each) with 5 μg Matrix-M adjuvant (Novavax, AB, Uppsala, SE). Serum was collected for analysis on study Day 21, one week after the 2nd dose.

For the booster study, female BALB/c mice (N = 10 per group, 200 mice total) were immunized by intramuscular (IM) injection with two 1 µg doses spaced 14 days apart (study day 0 and 14) of Prototype (control) or Prototype + Omicron BA.5 (0.5 µg each) with 5 μg Matrix-M adjuvant. A booster (3^rd^ dose) of 1 µg Omicron XBB.1.5 or XBB.1.16 with 5 μg Matrix-M adjuvant was administered on Day 47 (approximately 1 month post 2nd dose). Sera and spleen were collected 2 weeks after the booster dose on day 61 to evaluate antibody and cellular responses.

For all mouse studies, animals were randomly assigned to groups as they were removed from shipping cages.

### Nonhuman primate study design

Female and male rhesus macaques (*Macaca mulatta*, N = 5 per group; N = 15 total), 3–11 years old and weighing 3–9 kg at study initiation, were obtained from a SNPRC specific pathogen free (SPF) colony and/or Envigo (Alice, TX). Animals were randomly assigned to groups based on similar age and sex distribution across the groups. NHPs were immunized by intramuscular injection (0.5 mL) with the human dose level: 5 µg NVX SARS-CoV-2 Prototype (control) or Omicron BA.5 variant rS vaccines adjuvanted with 50 µg Matrix-M administered as monovalent, or bivalent prime/boost on days 0 and 21 (primary series). A booster consisting of Omicron XBB.1.5 rS with 50 µg Matrix-M was administered at week 35 (Day 246). Sera were collected prior to the boost on Day 210, as well as 2 weeks post boost on Day 260. Peripheral blood mononuclear cells (PBMCs) were also collected on Day 260.

### Pseudovirus neutralization

SARS-CoV-2 pseudoviruses were generated using a lentivirus platform adapted from Crawford, 2020^32^. Briefly, backbone and helper plasmids, including Wuhan-Hu-1 spike, were obtained from BEI Resources. Additional variants were synthesized in pcDNA3.1 (GenScript) using the appropriate spike protein sequence from the EPICoV database. All spike protein sequences included a deletion of the cytoplasmic tail. HEK293T cells were seeded one day prior to transfection, incubated at 37 °C overnight, and transfected when the cellular monolayer was 60–75% confluent. The transfection uses a cationic-lipid delivery system such as Lipofectamine 3000 (Thermo Fisher) or JetPrime Optimus (Polyplus) with a set of plasmids encoding: a lentiviral backbone, a dual reporter plasmid expressing both luciferase and Zs green, a plasmid expressing SARS-CoV-2 spike (such as Wuhan-Hu-1, Omicron BA.5, and BQ.1.1) and a plasmid expressing other HIV proteins for pseudovirion formation. Then, 48 h following transfection, supernatants were collected, centrifuged, and filtered through a 0.45 µm filter to obtain a pseudovirus stock. Commercial pseudovirus for Omicron XBB.1.5, XBB.1.16, XBB.2.3, and EG.5.1 were obtained from eEnzyme and incorporated only a luciferase reporter gene for detection of pseudoviral entry. Aliquots of pseudovirus stock were stored at − 80 °C. All work with pseudovirus was performed in a Biosafety Level 2 laboratory and approved by our Institutional Biosafety Committee.

Each newly produced lot or new shipment of pseudovirus, if commercially obtained, was titered under assay conditions to determine the working dilution to target an RLU of 100,000 prior to testing serum. The pseudovirus neutralization assay was then performed using a HEK293T cell line stably expressing hACE2 (HEK293T/ACE2 obtained from Creative Biogene). Serum samples were heat-inactivated by placing in a 56 °C water bath for 30 min, followed by cooling to 4 °C immediately. Serum samples were serially diluted three-fold in reduced serum Opti-MEM starting at a 1:20 or 1:50 dilution in a 96-well tissue culture plate. Fifty microliters of SARS-CoV-2 Pseudovirus stock (corresponding to 100,000 RLU, range from 50,000–250,000) was then added to each well, followed by incubation at 37 °C for one hour. Then, 2.0 × 10^4^ HEK293T/hACE2 cells in 100 µL of HEK293T cell culture medium (DMEM without phenol red + 5% FBS + 1% Penicillin + streptomycin + glutamine) containing 1.25 µg/mL puromycin were added to the wells, followed by incubation for 72 h at 37 °C. After incubation, 50 µL BrightGlo Luciferase Substrate (Promega) was added to each well. Plates were incubated for 5 min at room temperature without ambient light. Viral entry into the cells was determined by measuring the luminescence with a SpectraMax iD3 microplate reader. Pseudovirus neutralizing antibody titer of the serum was determined through the absence or reduction of luminescence in a well. Data were analyzed and neutralization curves were generated in GraphPad Prism for each sample; 50% pseudovirus neutralization titers (pVN_50_) and 50% inhibition dilution (ID50) were calculated using 4-parameter curve fitting. No-serum wells were present on each plate along with at least one positive and negative monoclonal antibody for each pseudovirus tested.

A similar pseudovirus neutralization assay, validated for testing human samples (for Ancestral, Omicron BA.5 and XBB.1.5 strains), was utilized as fit-for-purpose for testing NHP samples. This method is similar to the method used for the mouse studies except the input virus targeted an RLU of 50,000 (range 10,000–300,000) per well and diluted in infection medium containing Dulbecco’s Minimal Essential Medium (DMEM) and 2% heat inactivated fetal bovine serum (FBS), test serum and pseudovirus was incubated for 2-h; 10,000 cells/well were used for the assay. Serum dilution series started at 1:10, which was reported as 1:20 after addition of virus. Luciferase readout was performed from 15 min after addition of luciferase reagent up to 60 min, followed by data calculation using Softmax 4-parameter curve fit.

### Antigenic cartography

Pseudovirus neutralizing antibody titers in mouse sera were subjected to antigenic cartography analysis to visualize antigenic diversity (reviewed in^33^). Antigenic cartography maps were constructed using Cartography software available at acmacs-web.antigenic-cartography.org. Pseudovirus neutralizing titers in sera collected on Day 21 (1 week after the two-dose primary immunization) and Day 61 (2 weeks after the booster) were input into the software to construct a SARS-CoV-2 antigenic map. SARS-CoV-2 antigens are depicted as circles and sera are indicated as small squares. Each grid square represents one antigenic unit representing a two-fold change in titers. The antigenic distances among Prototype, Omicron XBB.1.5, XBB.1.16, and XBB.2.3 variants were calculated after the primary series and after the booster dose. Antigenic Distances were converted to fold-differences.

### Cellular assay

For ICCS assay of murine splenocytes, cells were cultured in a 96-well U-bottom plate at 1–2 × 10^6^ cells per well. The cells were stimulated with NVX-CoV2373 or the indicated SARS-CoV-2 variant spike protein. The plate was incubated 6 h at 37 °C in the presence of BD GolgiPlug™ and BD GolgiStop™ (BD Biosciences) for the last 4 h of culture. Cells were labeled with murine antibodies BV650 CD3 (Clone 145-2C11, 1:25), APC-H7 CD4 (Clone GK1.5, 1:25), FITC CD8 (Clone 53–6.7, 1:25), Alexa Fluor 700 CD44 (Clone IM7, 1:50), and PE CD62L (Clone MEL-14, 1:50) (BD Pharmingen, CA), and the yellow LIVE/DEAD® dye (1:300). After fixation with Cytofix/Cytoperm (BD Biosciences), cells were incubated with PerCP-Cy5.5-conjugated anti-IFN-γ (Clone XMG1.2, 1:50), BV421-conjugated anti-IL-2 (Clone JES6-5H4, 1:100), PE-cy7-conjugated anti-TNF-α (Clone MP6-XT22, 1:800), and APC-conjugated anti-IL-4 (Clone 11B11, 1:100) (BD Biosciences). All stained samples were acquired using an LSR-Fortessa flow cytometer or Symphony A3 (Becton Dickinson, San Jose, CA) and the data were analyzed with FlowJo software version 10 (Tree Star Inc., Ashland, OR). Data shown were gated on CD44^hi^ CD62L^low^ effector CD4 + T cell population.

For ICCS assay of NHP PBMCs, the cells were thawed and rested at 37 °C overnight. The cells were then stimulated as described above with NVX-CoV2373 or the indicated variant protein. Cells were labeled with human/NHP antibodies BV650-conjugated anti-CD3 (Clone SP34-2, 1:10), APC-H7-conjugated anti-CD4 (Clone L200, 1:10), APC-conjugated anti-CD8 (Clone RPA-T8, 1:10), and the yellow LIVE/DEAD® dye (1:300) for surface staining; BV421-conjugated anti-IL-2 (Clone MQ1-17H12, 1:25), PerCP-Cy5.5-conjugated anti-IFN-γ (Clone 4S. B3, 1:10), and PE-cy7-conjugated anti-TNF-α (Clone Mab11, 1:50) (BD Biosciences) for intracellular staining. Data shown were gated on CD4 + T cell population.

### Statistical analysis

Mann–Whitney *U* Tests (two-tailed) were used when determining statistical significance of differences between two groups, and Kruskal–Wallis Multiple Comparisons Test was used when comparing differences among three groups. GraphPad Prism 9.0 software (La Jolla, CA) was used to conduct statistical tests, calculate geometric mean titers (GMTs) and 95% confidence intervals (95% CIs), and plot data.

## Data Availability

The datasets generated during and/or analyzed during the current study are available from the corresponding author on reasonable request.
